# Experiences of the Covid-19 pandemic: From the perspective of an MSW student

**DOI:** 10.1177/1473325020981092

**Published:** 2021-03

**Authors:** Tina Hugo

**Affiliations:** Lyle S. Hallman Faculty of Social Work, Wilfrid Laurier University, 120 Duke St W, Kitchener, Ontario, N2H 6P6, Canada

**Keywords:** Practicum, reflexivity, self-concept, Covid-19

## Abstract

This reflexive essay offers an uncensored narrative and delves deep into the life of one MSW student during the Covid-19 pandemic. Five themes were uncovered through intense reflexive based practices and these included: parenting and food insecurity, silencing of the voice, grieving the practicum placement, the white gaze and a reduced empathetic capacity. Ultimately, meaning making is achieved through unsettling the concept of the self, identifying and exposing vulnerability and deflowering the power dynamics operating within Canada.

## Introduction

This reflexive essay will offer a bird’s eye view into my experiences as a Masters of Social Work (MSW) student during the Covid-19 pandemic. Through reflection, I will highlight my response to the unfolding of the pandemic but also through the process of reflexivity, I will delve deeper to uncover my embedded assumptions, underlying biases, embodied reactions, cultural positioning and the power dynamics in operation (Badwell, 2016; Rossiter, 2007). Five themes that surfaced were parenting and food insecurity, the silencing of my voice, grieving the practicum placement, the white gaze and a reduced empathetic capacity. My attempt at writing an uncensored narrative has been deeply unsettling and a difficult experience. I have learned that my sense of security throughout the Covid-19 virus and pandemic has likely been due to my lack of intersecting identities, my privilege. This reflexive essay weaves in my unsettled identity, my vulnerability and it examines the power dynamics that uphold my privilege.

To begin, I am a thirty-seven year old Caucasian woman. I identify as able-bodied, cis-gendered and a heterosexual individual. I am married to an able-bodied, cis-gendered male and we have two sons aged four and six years. I work part-time as a Health Navigator within a chronic disease and dialysis unit that is located in a rural hospital which serves two nearby towns and seven reserves. I live in Fort Qu’Appelle which is located in southern Saskatchewan and is on Treaty Four Territory. My husband is a Constable with Canada’s federal police service, the Royal Canadian Mounted Police (RCMP) and has been so for the past eleven years. Fort Qu’Appelle is our third posting and very near to our home of origin. Currently, I am in my last year of a distance MSW program at Wilfrid Laurier University which is located in Ontario.

## Parenting and food insecurity

As the Covid-19 pandemic unfolded in Saskatchewan, grocery stores posted signs in their windows and doorways which allowed only one child per parent in the stores. In general though, children were encouraged to stay home. Those few times when I would need to take my two young sons with me to purchase groceries, I felt ashamed. Looking around the store, my sons were the only children walking the aisles. Patrons and clerks looked mortified if one of my sons came too close to them. They would grimace and back away from us. It felt like society was repulsed by children. I wished I could leave my sons with family but this wasn’t possible due to Saskatchewan’s Covid-19 recommendations to stay only within primary family units. Shopping with my sons during the Covid-19 pandemic reminded me of being a young girl and shopping for groceries with my mother and my siblings.

When my mother received her social assistance check or her child tax benefits, my siblings and I would get all bundled up to go to the grocery store. Hand-in-hand, we would walk several blocks to get to the grocery store and once there, people would stare at my nineteen year old mother and her three young children. The patrons and clerks didn’t stare lovingly or with kindness at us. My mother walked through the store with her head held high and pretended like she didn’t see the people watching us. It was always a joyous occasion for me because it meant we would eat delicious food for a few days, maybe even a week. My mother never said she was ashamed on these grocery outings but I always felt that she was. She would become tense and rushed. As children, we wanted to take our time and explore all of the treats.

My identity as a woman is intertwined with that of being a mother. Being a working mother who can drive to the grocery store whenever I want to is a privilege. Being able to feed my children food that is healthy and nutritious seems unfair when I imagine all of the children whom are going hungry. While, access to certain products and foods was briefly thwarted by a food insecurity issue in Canada due to Covid-19, my family didn’t go without. I knew that I could stockpile if I needed to. My privilege affords me the luxury that I don’t have to wait for a certain day of the month to buy groceries. Prior to the Covid-19 virus, my social position as a thirty-seven year old mother granted me a different experience of grocery shopping than that of my mothers’. Almost always, I received kindness and stares of adoration from patrons and clerks. In a small way, through the Covid-19 virus, I connected to the shame that a young mother on social assistance might experience while grocery shopping. While, having to hide my children from society wounded me, it also humbled me.

## The silent voice

When Covid-19 was announced to the world, I was half way through my foundational practicum. This practicum placement was in an Indigenous harm reduction site called Miko Mahikan Red Wolf. My last day of practicum occurred on March 3, 2020. As I reflect back, this was a blurry and confusing time. I vividly remember the staff at my practicum placement making comments that they thought everyone was overreacting to Covid-19. From hearing my coworkers minimize the impact of Covid-19 (which wasn’t officially declared a pandemic yet), I wondered if I was one of those people whom were overreacting. Being a practicum student, I doubted myself. I doubted my thoughts and even my feelings. Conversations about Covid-19 were tense and brought up feelings of fear for me. In the safety of my home, my husband and I could talk openly about our fears of the virus. Anywhere outside of my home, I began to monitor and eventually silence myself. Silencing my voice was new but somehow it was also eerily familiar.

Only one other time have I silenced my voice. I was eight years old and in grade three. In the span of two months time, my closest family member passed away and my parents separated. My mother left me and my sister to live with an estranged uncle and his blended family. It was during this time that I stopped talking all together. I would nod my head in agreement, or I would offer a toothless smile, but I wouldn’t talk. During the beginning of the Covid-19 pandemic, I didn’t stop talking all together, but I felt myself nodding and smiling. I camouflaged my thoughts and feelings by physically and verbally agreeing with my coworkers. My body was agreeing with people but my mind wasn’t. The anxiety would swell up inside of me, like a tick full of blood. Silencing myself allowed the anxiety to grow inside of my body. I felt immensely conflicted with being a “good” social worker, whom would speak up, versus, just trying to get by moment to moment. In my practicum placement, I was seeking authentic and rich relationships with my coworkers, but upon reflection, the Covid-19 virus wouldn’t allow for such vulnerability. Looking back, I was clouded with fear, confusion and uncertainty. Having this experience, I have come to realize how silencing myself inscribes silence in other groups as well. At a time, when I needed to be open and share in order to heal emotionally, I reverted back to silence.

As I continued to silence myself, I became detached from my feelings in relation to the Covid-19 pandemic. After seven weeks, I no longer felt the anxiety well up inside of me. I felt nothing, I was numb. Then, I noticed a tightness in my chest one night while I was watching the news. I had been watching two hours of CTV news coverage and I was completely transfixed on Covid-19 in Italy. Eventually that tightness in my chest turned into a chest infection where the symptoms mirrored Covid-19’s symptoms. Once again, the anxiety came flooding back and I realized that self-preservation is a short term solution to a long term problem.

## Grieving the practicum placement

March 3, 2020 was my last shift at my foundational practicum, but I didn’t know it at that time. As the practicum was terminated (but deemed completed), I wasn’t able to receive any sort of a good-bye from the individuals that I worked with. I didn’t realize how attached I became to having a positive ending. Looking back, I see that I became fixated on a ceremonious ending with gift giving, hugs and good bye’s.

I was so consumed with the MSW program and practicum that I was unable to truly connect to the society that was crumbling around me. People were loosing their health, their lives, their jobs and their homes. Here I was grieving a practicum placement. It was at this time that I realized just how much power the government occupied. For the first time, I felt that I was at the mercy of a system. Just as Canada began making rapid decisions, so too did those in power at Wilfrid Laurier University. I received email after email from the University highlighting their plan to support MSW students. I watched the Prime Minister, Justin Trudeau comfort Canadians in much the same way. All the while, in Saskatchewan the pandemic hadn’t fully materialized. I felt helpless, confused and I wondered, “was this really happening?” Suddenly, I understood that my MSW program offered my mind a quiet place to retreat. Grieving the practicum placement can be viewed as a site of privilege.

## The white gaze

As I stand in line to order a coffee at my local coffee shop, my gaze falls on an employee who is many months pregnant. She is wearing a light blue mask and is taking customer’s orders. My gaze washes up and down this woman and I understand that not all families are as fortunate as mine. Her gaze meets mine and my eyes quickly dart away. I feel culpable that this young woman has had to sacrifice her health to give me a coffee. I wonder, “does she feel abandoned by our government and by our society?” Does she blame me? Then, I wonder how racialized people and other marginalized people feel? I question the sacrifices they might have made. Have they lost a family member, a job, a home, or even a sense of safety? I have lost none of these. As “Black Lives Matter” surges around me, I understand how my white face has sheltered me.

My gaze returns to the woman, she reminds me that my husband was able to take on more family responsibility (working night shifts and homeschooling during the day). She reminds me that our privilege has provided us with workplaces that have accommodated our family’s needs. In this moment, I know that my gaze is neither innocent nor vulnerable. I realize that while I am grateful for my privilege, I am also terrified of losing it.

## Reduced empathetic capacity

My embodied experience is that in my little rural town it is safe, and outside of my town it is unsafe. As Covid-19 ravaged on, a collective consciousness of fear swooped in on Canadian society. I feared opening up Canada’s border to the United States. I imagined restaurants and our national parks being flooded with people infected with the Covid-19 virus. Canada’s news stations highlighted story after story about the devastation that the United States was experiencing due to the Covid-19 virus. I pictured Canada’s universal healthcare being consumed with just managing the virus. Feelings of protectionism arose within me. Up until this point, my rural town and southern Saskatchewan region had experienced very low rates of positive Covid-19 cases and for me, it felt like the sickness was out there and located in the “other”. I couldn’t relate to the individuals who were sick and dying. I also couldn’t empathize with the families of the sick and dying.

I want to blame the media for manufacturing this fear-based collective consciousness. I want to believe that my reduced empathetic capacity for those individuals and communities struggling with the Covid-19 virus is located within a collective consciousness of fear. But, what if my reduced empathetic capacity is because of my privilege? Having never known what it feels like to be a marginalized person in society has afforded me refuge and this refuge has largely been invisible to me.

## Conclusion

Through writing about my experiences as an MSW student during the Covid-19 pandemic, I have learned that being a white, middle class, able-bodied, heterosexual and married woman has afforded me a sense of security during these trying times. This narrative has been my attempt to be vulnerable and forthcoming of my experiences. Outing my privilege doesn’t valorize me and it doesn’t change the systemic barriers that Canada embodies. In no way does my openness undo my privilege, but it does expand my container, so that I am able to hold more, see more and feel more.
